# Sex Ratio Meiotic Drive as a Plausible Evolutionary Mechanism for Hybrid Male Sterility

**DOI:** 10.1371/journal.pgen.1005073

**Published:** 2015-03-30

**Authors:** Linbin Zhang, Tianai Sun, Fitsum Woldesellassie, Hailian Xiao, Yun Tao

**Affiliations:** Department of Biology, Emory University, Atlanta, Georgia, United States of America; University of California Davis, UNITED STATES

## Abstract

Biological diversity on Earth depends on the multiplication of species or speciation, which is the evolution of reproductive isolation such as hybrid sterility between two new species. An unsolved puzzle is the exact mechanism(s) that causes two genomes to diverge from their common ancestor so that some divergent genes no longer function properly in the hybrids. Here we report genetic analyses of divergent genes controlling male fertility and sex ratio in two very young fruitfly species, *Drosophila albomicans* and *D*. *nasuta*. A majority of the genetic divergence for both traits is mapped to the same regions by quantitative trait loci mappings. With introgressions, six major loci are found to contribute to both traits. This genetic colocalization implicates that genes for hybrid male sterility have evolved primarily for controlling sex ratio. We propose that genetic conflicts over sex ratio may operate as a perpetual dynamo for genome divergence. This particular evolutionary mechanism may largely contribute to the rapid evolution of hybrid male sterility and the disproportionate enrichment of its underlying genes on the X chromosome – two patterns widely observed across animals.

## Author Summary

Millions of species live on Earth, thanks to an evolutionary process that splits one species to two or more new species. The formation of new species is benchmarked by the evolution of reproductive isolation (RI) such as hybrid sterility between new species. The fundamental question of how RI evolves, however, remains largely unknown. In a pair of very young fruitfly species, we localized six loci expressing dual functions of hybrid male sterility (HMS) and sex ratio distortion, implicating an evolutionary causal link between these two traits. The rapid evolution of HMS widely observed across animal taxa can be attributed to the rapid evolution of genes controlling sex chromosome segregation. All genes in a genome are not equal. This study suggests that conflicts among various parts of a genome might confer strong evolutionary pressure—a mechanism that has hitherto been regarded as rare and could actually be more ubiquitous than currently appreciated.

## Introduction

Intrinsic reproductive isolations (RI) between two newly evolved species can take the forms of hybrid male sterility (HMS), hybrid female sterility (HFS) and hybrid inviability (HI), all manifestations of genetic incompatibilities between two genomes [1]. Speciation genetics studies typically start with genetic analysis of divergent reproductive traits between two species. Numerous genes underlying interspecific divergence have been identified [2,3], but they cannot be automatically qualified as “speciation genes” because some interspecific divergence may have evolved only after speciation was complete. The identification of genes underlying HMS, HFS and HI—also called Dobzhansky-Muller incompatibility (DMI) genes—by themselves, even with their biological functions well understood, can rarely answer which DMI genes are involved in establishing the initial RI, and what adaptive phenotypes of these genes are responsible for their fixations in one but not the other lineage [2]. Thus the evolutionary mechanism(s) for evolving DMI at the initial stage of speciation still remains a mystery.

Nevertheless, two patterns have emerged from extensive speciation genetic studies in the last three decades. The first is the “faster male” evolution in that HMS evolves at a rate an order of magnitude higher than HFS and HI [4], presumably caused by sexual selection [5]. The second is the “large X” evolution in that HMS genes are enriched on the X chromosomes [6–8], presumably caused by more efficient fixation of mutations on the X than on autosomes [9]. However, sexual selection would also make hybrid ZZ males more likely to be sterile than hybrid ZW females, but this prediction is not supported by empirical observations [4]. Similarly, efficient selection of X-linked genes would also predict the “large X” pattern for the HI genes but no empirical support has been garnered either [10]. Thus, neither the “faster male” nor the “large X” pattern has been sufficiently accounted for by any evolutionary theories as well as the associated empirical evidence.

The above two patterns can be better explained by the “conflict theory” in that genomic divergence is driven by selfish genes, prominently by sex ratio distortion (SRD), also called sex chromosome meiotic drive [11–13]. Meiotic drive distorter breaches Mendel’s first law of genetics by gaining more than 50% transmission while quenching its homolog’s share in the gene pool of next generation. The distorter, however, does not commit suicide because of the tightly linked insensitive responder, while its homolog is linked to the sensitive responder. Meiotic drive is generally harmful to a genome, thus suppressors to silence the distorter are under strong selection to evolve and make the meiotic drive cryptic [14]. A tight linkage between the distorter and the responder is a key requirement for a meiotic drive system to evolve [15]. This prerequisite is readily satisfied on the two heteromorphic sex chromosomes, between which recombination is generally absent. Sex chromosome meiotic drive manifests as unequal sex ratio. For a typical XY male, the optimum sex ratio is all females for the X-linked genes but all males for the Y-linked genes, and 50% females for all autosomal genes. Therefore, the optimum sex ratios are at odds from the perspectives of various portions within a genome [16]. If SRD arises repeatedly on the X chromosome, counter evolution on the Y and the autosomes is anticipated, so much so that the SRD operates as a perpetual dynamo for genome evolution and bouts of this distortion-suppression process eventually lead to speciation [13]. The “conflict theory” can readily account for the “faster male” evolution because SRD occurs in XY male, and the “larger X” evolution because this chromosome contributes about half of the genetic changes in the evolution caused by SRD [13]. The “conflict theory” also predicts “faster female” in ZW females [4], and a faster pace of RI evolution in taxa with heteromorphic sex chromosomes than those without.

The best evidence for the “conflict theory” comes from two HMS genes with dual functions of SRD and HMS: *tmy* mapped between *D*. *simulans* and *D*. *mauritiana* [17], and *Ovd* identified between *D*. *pseudoobscura* USA and *D*. *p*. Bogota [18]. However, these SRD systems could have evolved after speciation. Many other HMS genes are also mapped in these species but they do not have the SRD phenotype [19,20], so are almost all the other known HMS genes across all taxa. Therefore HMS seems to have evolved by mechanisms generally unrelated to SRD. On the other hand, absence of SRD phenotypes in hybrids can be explained by the absence of idiosyncratic genetic background required for SRD expression, gene silencing and loss of function in cryptic SRD systems, or sterility of hybrids. Indeed, there might be an intrinsic difficulty to test the “conflict theory” because the SRD expression is usually transient.

**Fig 1:**
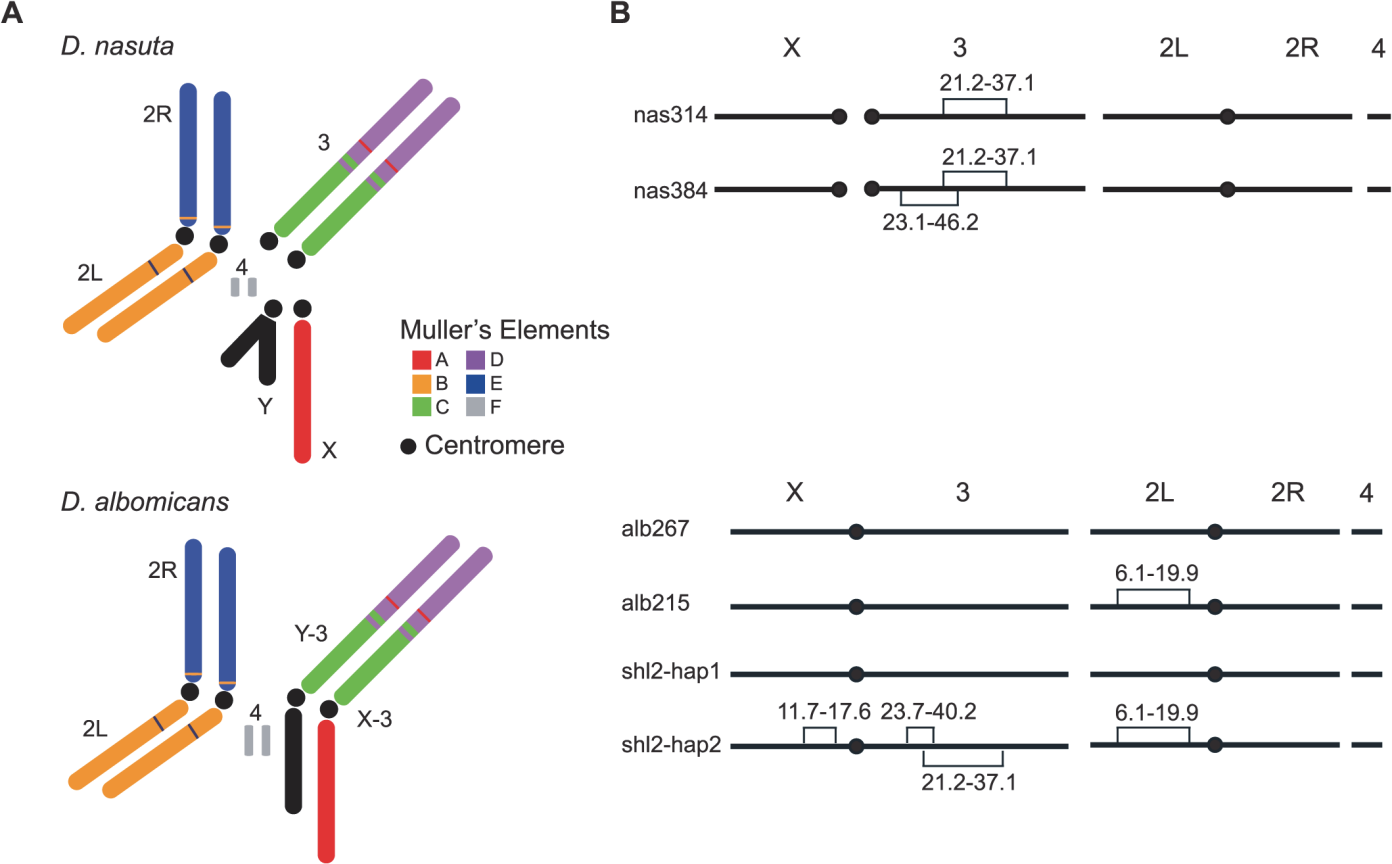
Key stocks of *D*. *albomicans* and *D*. *nasuta* used in this study. (A) Karyotypes of these two species. Robertsonian fusions between the X (Y) and the 3^rd^ chromosomes in their ancestor led to a pair of new sex chromosomes X-3/Y-3 in *D*. *albomicans*. Muller’s elements are assigned to chromosomal arms, along with the translocations detected by molecular markers and genetic linkage from this study (S1 Dataset and S1 Table). (B) Stockes used in this study. Shown are fixed inversions and the associated breakpoints for true-bred stocks (alb267 and alb215) derived from alb2, and nas314 and nas384 from nas3, as well as the segregating inversions (shl2-hap1 and shl2-hap2) in shl2. The polytene sequence was determined according to the standard map made from alb267 (S2 Dataset). The centromere position on the “dot” chromosome 4 is not determined.

We reasoned that an ideal empirical system for identifying the *bona fide* “speciation genes”, to test the “conflict theory” or any other theories of speciation for that matter, would be a pair of species at the very incipient stage of speciation, when the HMS just starts to evolve and is directly responsible for establishing the initial RI. Two *Drosophila* species, *D*. *albomicans* and *D*. *nasuta*, appear to be such a system because of their young age of ∼120 kyrs [21]. *D*. *albomicans* is distributed from Okinawa of Japan through South China, Indochina to Northeast India, while *D*. *nasuta* is found in East Africa, Madagascar, Seychelles, Mauritius, Sri Lanka, and the India subcontinent [22]. These two species are not distinguishable in morphology but have distinct karyotypes. *D*. *nasuta* has the ancestral karyotype (2n = 8), but the acrocentric 3^rd^ chromosomes are fused to the X and Y to form a pair of new sex chromosomes (X-3/Y-3) in *D*. *albomicans* (2n = 6) (Fig. 1). There is almost no pre-mating isolation between these two species [23], and only weak hybrid breakdown was observed in the hybrids of advanced generations [24,25]. SRD is expressed in the F1 males produced by females of certain strains of *D*. *albomicans* crossed to *D*. *nasuta* males [24–27]. The sex ratio (k, proportion of female) is skewed (k = ∼0.90) if the *D*. *albomicans* strains are from Okinawa but normal (k = ∼0.50) if the strains are from Southeast Asia. There is an apparently increasing cline of SRD strength from SE Asia to Japan [24,26].

The “conflict theory” will be strongly supported if most HMS genes have contemporary or historical functions of SRD. The incipient species pair *D*. *albomicans* and *D*. *nasuta* qualifies as an excellent empirical system for testing the “conflict theory” because both SRD and HMS are expressed in their hybrids. For that, we mapped the genes of HMS and SRD simultaneously through three QTL mappings and multiple lines of introgressions. These genes are polymorphic within *D*. *albomicans*. A majority of the genes controlling both traits are colocalized to the same six regions. These findings implicate a contemporarily active SRD system that may have an evolutionary causal link to hybrid male sterility, thus lending strong support to the “conflict theory” of speciation.

## Results

### The study system

For the genetic dissection of SRD and HMS in the species pair *D*. *albomicans* and *D*. *nasuta*, we first constructed three inbred lines, two from *D*. *albomicans* (alb2—Okinawa; shl2—NE India) and one from *D*. *nasuta* (nas3—Mauritius) (Materials and Methods). We then surveyed male and female fertilities of these stocks and various F1 genotypes with standard methods, in which single males or females were mated to three virgin testers for 7 days and the progeny size was regarded as the fertility of the tested males or females (see Materials and Methods for details). By the standard methods, all interspecific F1 hybrids appeared to have normal or nearly normal fertility. As expected and consistent with previous studies [24–27], SRD was expressed in the F1 males from alb2♀ × nas3♂ (k = ∼0.9) but not in the F1 males from shl2♀ × nas3♂ and most of the other crosses (k = ∼0.5) (S1 Fig).

Unfortunately, all three inbred stocks are still polymorphic for chromosomal inversions, thus are not ideal for genetic mapping, a major goal of this study. Two true-bred stocks, alb267 and alb215, were then extracted from alb2 with the help of molecular markers (S1 Dataset), so were nas314 and nas384 from nas3. However, we failed to construct inversion-free stocks from shl2, presumably due to recessive sterile mutations locked in the inversions on the two haplotypes (shl2-hap1 and shl2-hap2) (Fig. 1; Materials and Methods). Chromosomes from alb2 or shl2 are not homosequential to those of nas3, thus regions in and around the inversions are not accessible to genetic mapping. However, alb267 and shl2-hap1 are homosequential and have the same standard polytene sequence (S2 Dataset).

### HMS and SRD found between *D*. *albomicans* and *D*. *nasuta*

**Fig 2:**
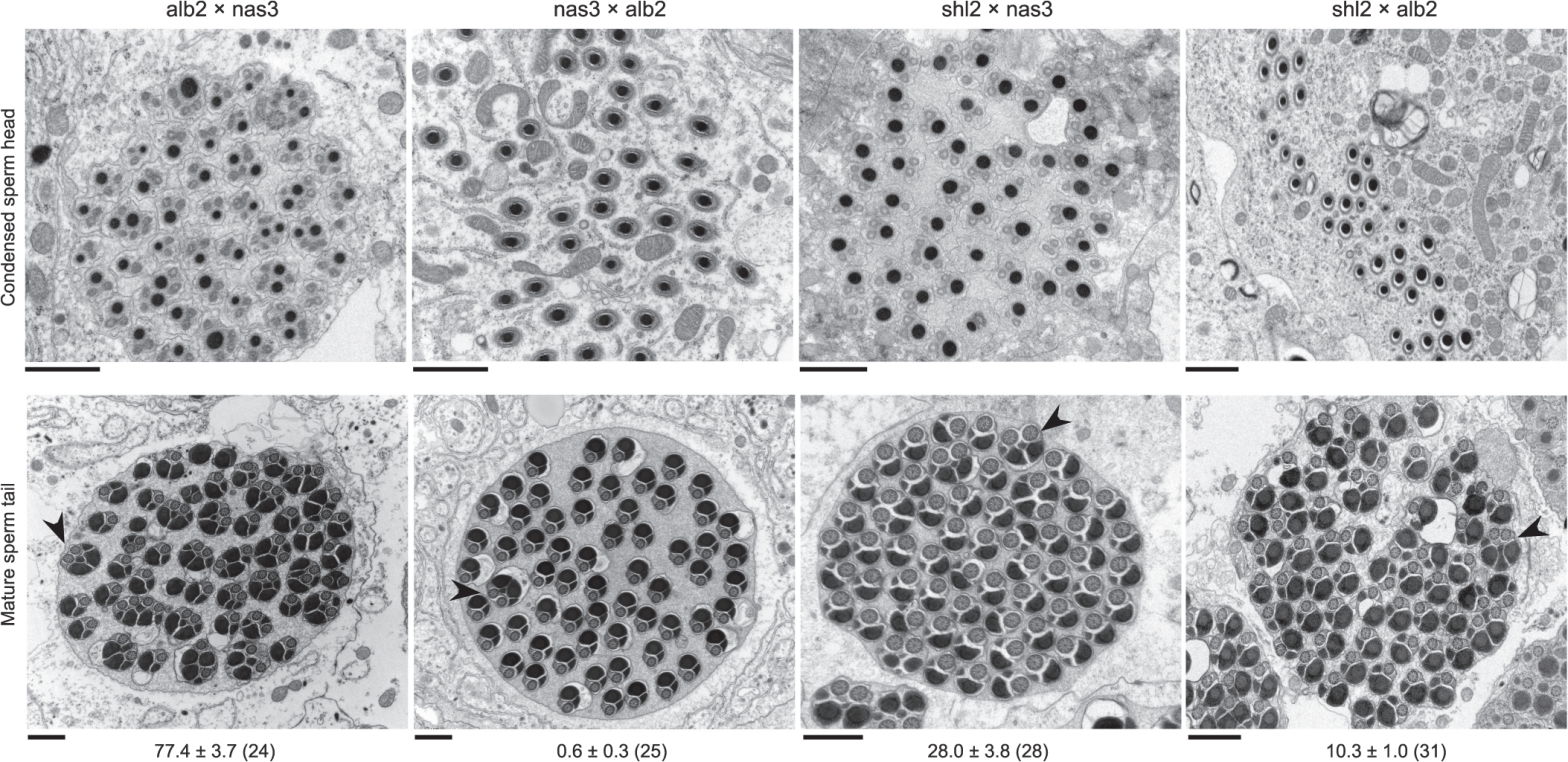
Abnormal spermatid indicating HMS. Cross sections of condensed sperm heads (upper row) and mature spermatid tails (lower row) in the F1 males from three interspecific (first three columns) and one intraspecific control (rightmost column) crosses (♀ × ♂). All sperm heads were normal, but frequent twin tail fusions (arrow heads) were counted in all but the F1 males from nas3 × alb2 (Mean ± s.e.m. with the numbers of spermatid bundles examined in parentheses under the representative TEM images. P < 0.05 for all pairwise comparisons, ANOVA and TukeyHSD). All scale bars are 1 μm.

The standard test is not powerful enough to detect HMS between these two species. Some subtle abnormalities in spermatogenesis can be revealed by cytological methods. We thus used transmission electron microscopy (TEM) to examine spermatogenesis in the F1 males from the interspecific crosses alb2♀ × nas3♂, nas3♀ × alb2♂ and shl2♀ × nas3♂, as well as that from the intraspecific cross shl2♀ × alb2♂ (Figs. 2, S2). Sperm head development was normal in all the F1 males examined, even those expressing SRD. In contrast, sperm head condensation during spermatogenesis is disrupted in two well studied meiotic drive systems in *Drosophila* [28,29]. However, pairs of sperm tails were often fused as a characteristic abnormality after the stage of sperm head condensation in many of these males examined. These twin tail fusions were more frequent in the F1 males from alb2♀ × nas3♂ (77% of tails) than those from shl2♀ × nas3♂ (28%), suggesting severer HMS effects contributed by alb2 than shl2 alleles. Unexpectedly, frequent twin fusions (10%) were also seen in the F1 males from the intraspecific cross shl2♀ × alb2♂, tentatively suggesting that HMS has also evolved between these two strains of *D*. *albomicans* and possibly also a collateral effect of SRD evolution within the same species. But the intraspecific divergence needs further study with multiple strains of *D*. *albomicans*, a species with a very wide geographic distribution. On the other hand, no fusions were found in the hybrid F1 males from nas3♀ × alb2♂ in contrast to the F1 males from the reciprocal cross (alb2♀ × nas3♂), suggesting a lack of HMS loci residing on the X chromosome of nas3 and/or an enrichment of HMS loci on the X-3 chromosome of alb2. Thus, the TEM studies provide evidence that slight HMS has evolved between these two species.

**Fig 3:**
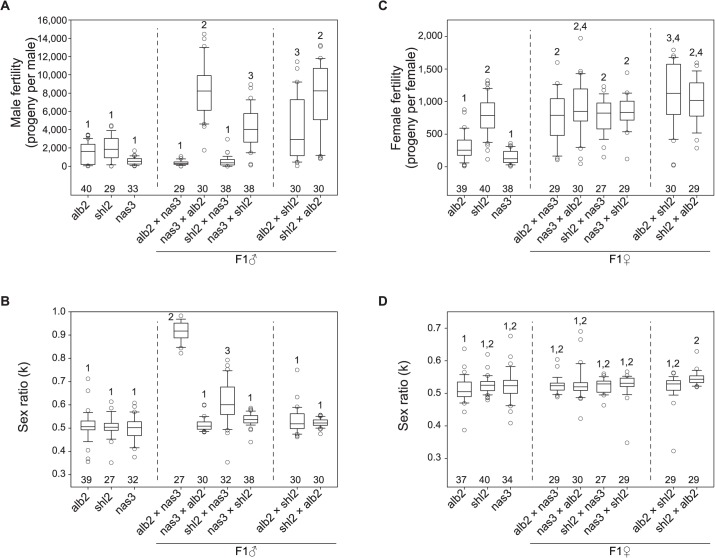
Polymorphism of HMS and SRD genes in *D*. *albomicans*. Males and females from inbred lines of *D*. *abomicans* (alb2, shl2) and *D*. *nasuta* (nas3) were tested for fertility and sex ratio using the exhaustive mating protocol, so were the F1 males and female from the crosses (♀ × ♂) among these three lines. Virgins from alb2 were used as testers throughout (Materials and Methods). Box plots of male fertility (A) and offspring sex ratio (B), female fertility (C) and offspring sex ratio (D). Phenotypes not sharing the same numerals (1–4) in each panel are significantly different (P < 0.05, ANOVA and TukeyHSD). The sample sizes are shown above the abscissa.

In order to further quantify the weak HMS observed above, we developed a novel, exhaustive mating protocol with the assumption that all of functional gametes can fertilize eggs, so the sperm can be “counted” as progeny size (S3 Fig, Materials and Methods). The results are summarized in Fig. 3. To interpret the data, we posit that there were three antagonistic effects working simultaneously in the tested flies: inbreeding depression, hybrid vigor and outbreeding depression. Inbreeding depression caused much lower fertility of both sexes of the inbred stocks alb2, shl2 and nas3, while hybrid vigor increased the fertility of the F1 males from both reciprocal crosses of shl2 × alb2 (Fig. 3A, C); outbreeding depression, *i*.*e*., DMI including HMS, brought down fertility in the F1 males from alb2♀ × nas3♂ and shl2♀ × nas3♂, but not in the F1 males from their respective reciprocal crosses (Fig. 3A). Somewhat consistent with the TEM studies, the fertility of the F1 males from alb2♀ × nas3♂ (mean ± s.e.m = 352 ± 46 offspring per male) was marginally worse than that from shl2♀ × nas3♂ (515 ± 91, 1-tail *t*-test, P = 0.058). Unlike males, fertility in hybrid females was largely not affected (Fig. 3C). The latter contrast is expected because HMS evolves much faster than HFS [4,30], and there might be only negligible HFS evolution between this pair of species at the very beginning of speciation.

Strong SRD (k = ∼0.92) was expressed in the F1 males but not the F1 females from alb2♀ × nas3♂, consistent with previous interpretation that the observed sex ratio skew is caused by SRD rather than by other mechanisms such as male killing [27]. But unexpectedly, weak SRD (k = ∼0.63) was also detected in the F1 males from shl2♀ × nas3♂ by the exhaustive mating protocol (Fig. 3B). Like HMS, the SRD of this genotype was not detected by standard method (S1C Fig). SRD expression might be affected by sperm storage or competition that must differ between these two mating test protocols.

In sum, both HMS and SRD genes are polymorphic within *D*. *albomicans* and their effects are often subtle and difficult to assay. The HMS effects are slight and roughly amount to inbreeding depression suffered in the inbred parental lines. The asymmetry of HMS effects in the F1 males from reciprocal crosses suggests that only a few HMS loci are present [31]. Genotypes with stronger SRD appear to have severer HMS, suggesting a possible connection between these two traits. The HMS and SRD genes might have been enriched on the X-3 chromosome, consistent with a general prediction of the “conflict theory” of speciation. In contrast, *D*. *nasuta* might have barely evolved any HMS effects on its X chromosome. The last inference might suggest a lack of SRD activity in *D*. *nasuta* since it was split from *D*. *albomicans*.

### QTL mapping

We took a quantitative trait loci (QTL) mapping approach to localize both the HMS and SRD loci divergent among the three chromosomal complements of alb267, shl2-hap1 and nas314 in three separate experiments (Exp1-3) (Materials and Methods). The mapping population of males in Exp1 was produced from crossing the F1 females from alb267 ♀ × shl2 ♂ to nas314 males. Although these males were F2, they actually had interspecific F1-like genetic constitution. The mapping is for genetic variations between these two strains of *D*. *albomicans* contributing to SRD and HMS between this species and *D*. *nasuta*. In contrast, the mapping populations in Exp2 and Exp3 were generated from backcrossing the F1 females from alb267 ♀ × nas3 ♂ and from shl2 ♀ × nas3 ♂, respectively, to the parental nas314 males. The latter two mapping populations had the backcross 1 (BC1) genetic constitution, and the mappings are for SRD and HMS genes divergent between *D*. *albomicans* and *D*. *nasuta*. All males of the three mapping populations were mating tested for fertility and sex ratio with standard method (S4 Fig; Materials and Methods).

**Fig 4:**
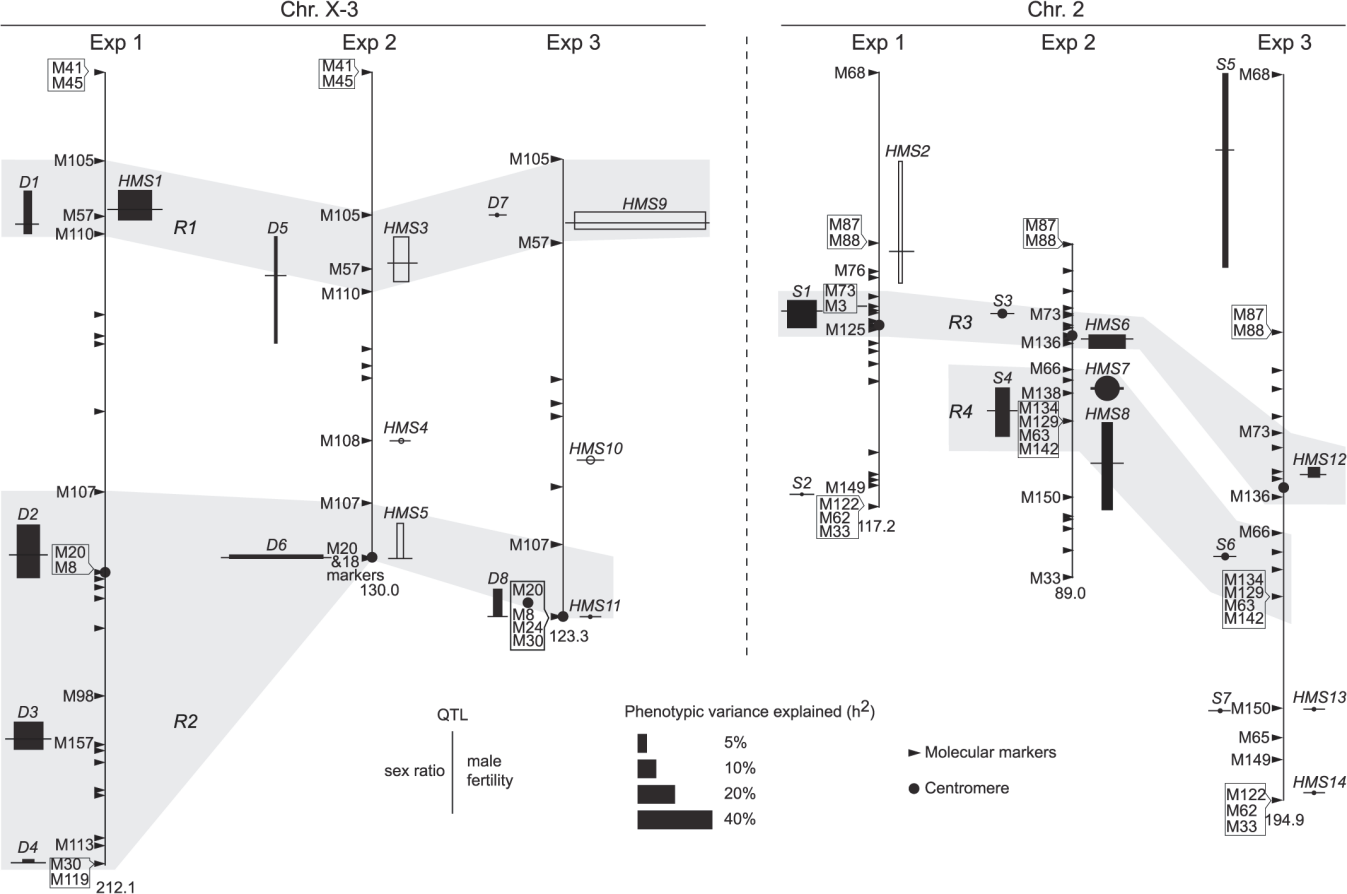
QTL detected from the three mapping experiments. Synopsized from S5 Fig and S1–S4 Tables. QTL of sex ratio and male fertility (T) are shown at the left and right of chromosomes, respectively. Linkage groups are shown to scale, with their total lengths shown at the distal ends. The names of some key markers are also shown (See S1 Table for complete dataset). “Good” QTL (rectangles) are those implicated by both CIM and MIM for sex ratio, or those by both T and at least one transformation of T (log_10_^(T+1)^ or binary) for fertility; otherwise the QTL are “tentative” (circles). For each QTL, the horizontal bar points to its most likely position on the map. The 95% confidence intervals and the phenotypic effects (measured as additive genetic variance *h* ^2^) of the “good” QTL are represented by the heights and widths of the rectangles, respectively. For “tentative” QTL, no 95% confidence intervals can be given, but their effects are proportional to the diameters of the circles. In Exp1, the alb267 alleles have positive effects (filled rectangle or circle) or negative effects (empty), as relative to the shl2-hap1 alleles. Similarly, the effects of the alb267 alleles are shown relative to the nas314 alleles in Exp2, so are the effects of shl2-hap1 alleles relative to the nas314 alleles in Exp3. *HMS7* is not drawn to scale because neither its location nor its effect is stable across the three mapping analyses (S5 Fig, S3 Table). Four regions (*R1–R4*) are highlighted because they contribute most of the *h* ^2^ in all three experiments (Table 2).

In QTL analyses, we measured male fertility simply as the raw offspring count (T). In addition, we also transformed T by log_10_^(T+1)^ or treated it as a binary variable (1 for fertile and 0 for sterile). These three treatments have different biological implications (See Materials and Methods). Consistent with polytene evidence, the third chromosome was almost totally refractory from recombination between alb267 and nas314, as well as between shl2-hap1 and nas314 (S1 Table). Not surprisingly, genetic divergence is low for HMS between alb267 and shl2-hap1 (*H* ^2^ = ∼20%, Exp1), so is it for SRD between shl2-hap1 and nas314 (*H* ^2^ = ∼13%, Exp3) (Table 1; S1 and S4 Tables). In contrast, genetic divergence is much higher for HMS between alb267 and nas314, as well as between shl2-hap1 and nas314 (*H* ^2^ = ∼42–92%, Exp2 and Exp3), and so is it for SRD between alb267 and nas314, as well as between alb267 and shl2-hap1 (*H* ^2^ = ∼78% in Exp2 and *H* ^2^ = ∼48% in Exp1, respectively) (Table 1; S2 and S3 Tables). For all mappings, a majority (∼66–100%) of the *H* ^*2*^ is additive (*h* ^*2*^) (Table 2). This is somewhat unexpected because the interactions between distorters and suppressors would suggest otherwise. The mapping results are incongruent for a few “tentative” QTL where the statistic inferences are not robust (S5 Fig). On the other hand, many QTL are “good” because they are stable with various data transformations and analytical methods (Materials and Methods). All QTL of total offspring (T) and sex ratio with their positions and phenotypic contributions are synopsized in Fig. 4. The nomenclatures of QTL imply their functions: distorter (*D*), suppressor (*S*) and hybrid male sterility (*HMS*).

**Table 1:** Genetic components of QTL mappings: Genetic variance (*H* ^2^) and additive genetic variance (*h* ^2^) in the three QTL experiments.

Experiment	Exp1			Exp2				Exp3			
	Fertility (T)	Log_10_(T+1)	Sex ratio	Fertility (T)	Log_10_(T+1)	Binary(T)	Sex ratio	Fertility (T)	Log_10_(T+1)	Binary(T)	Sex ratio
*H* ^2^ (%)	20.2	17.5	47.7	42.3	85.4	91.6	78.0	86.9	90.7	90.3	13.1
*h* ^2^ (%)	20.2	17.5	45.3	40.1	63.1	60.8	64.0	85.9	80.4	67.6	13.1
*h* ^2^/*H* ^2^ (%)	100.0	100.0	95.0	94.8	73.9	66.4	82.1	98.8	88.6	74.9	100.0

**Table 2:** Genetic components of QTL mappings: Percentage of *h* ^2^ contributed by the four regions *R1–R4*.

Experiment	Exp1			Exp2				Exp3			
	Fertility (T)	Log_10_(T+1)	Sex ratio	Fertility (T)	Log_10_(T+1)	Binary(T)	Sex ratio	Fertility (T)	Log_10_(T+1)	Binary(T)	Sex ratio
*R1*	90.1	88.6	9.9	20.2	16.8	14.5	2.8	88.6	84.8	70.4	5.3
*R2*	-	-	76.6	8.7	3.3	-	76.7	-	0.2	1.6	38.2
*R3*	-	-	10.4	49.6	45.6	21.1	8.1	7.7	13.3	25.6	-
*R4*	-	-	-	15.0	22.5	55.1	12.3	-	-	-	19.8
Total	90.1	88.6	96.9	93.5	88.2	90.7	100	96.3	98.3	97.6	63.3

Exp1 maps intraspecific genetic variations of SRD and HMS genes between the two *A*. *albomicans* complements alb267 and shl2-hap1. We found five “good” (*D1–D4*, *S1*) and one “tentative” QTL (*S2*) for sex ratio and two “good” QTL for male fertility (*HMS1*, *HMS2*). Except *D1* and *HMS1* that are colocalized, all the other QTL have only one phenotype. Exp3 maps SRD and HMS between shl2-hap1 and nas314 with two “good” (*D8*, *S5*) and three “tentative” QTL (*D7*, *S6*, *S7*) for sex ratio, and two “good” (*HMS9*, *HMS12*) and four “tentative” QTL (*HMS10*, *HMS11*, *HMS13*, *HMS14*) for HMS. Three pairs of loci (*D7*/*HMS9*, *D8*/*HMS11*, *S7*/*HMS13*) are colocalized, but the other five loci have only one phenotype. In these two experiments, the loci with only one phenotype might not genuinely have the alternate phenotype; or, more likely, the other phenotype falls short of detection because of the low *H* ^*2*^ and thus low power in QTL mapping. The latter interpretation is supported by introgression studies described in next section.

Since both SRD and HMS have high *H* ^*2*^ in Exp2, we expect that the mapping power would be more balanced between these two phenotypes. Indeed, all three “good” (*D5*, *D6* and *S4*) and one “tentative” (*S3*) sex ratio QTL from Exp2 are located to regions also harboring HMS QTL. The only exception is the tentative *HMS4* without SRD locus nearby (Fig. 4). QTL mapping is known for its lack of resolution, finer mapping is needed as we will show in the next section.

All together, four regions (*R1-R4*) harbor >90% of additive genetic variance (*h* ^2^) of SRD and HMS across the three QTL mappings with the only exception of SRD mapping in Exp3, where the “good” *S5* and “tentative” *S7* outside these four regions contribute 24.4% and 12.2% of *h* ^2^, respectively (Fig. 4, S4 Table). Because of the low *H* ^*2*^ for SRD in Exp3 (13%, Table 1), the robustness of detecting the SRD QTL from Exp3 is questionable; even worse in the case of *S5* because of the sparse markers nearby. Nevertheless, the overall colocalization of SRD and HMS suggests that these two traits have evolutionary connection. Because of the limited power and resolution of QTL mapping, more definite evidence can be reached by introgression studies as presented below.

### Introgression studies

We used a marker-assisted introgression approach to further increase mapping resolution of both SRD and HMS loci by testing the phenotypes of alb267 alleles in the nas314 background (Materials and Methods). Because SRD and HMS are oligogenic systems, the penetrance of the constituent elements depends on appropriate genetic context. Therefore the phenotypes of individual QTL can be best assayed by contrasting two introgression genotypes with and without the focal alb267 alleles (S6 Fig). In addition to the regions of *R1* (*D5*/*HMS3 = D1*), *R3* (*S3*/*HMS6 = S1*) and *R4* (*S4*), individual loci of *D2*, *D3* and *D4* in the *R2* region were also assayed after rare recombinants had been obtained on the 3^rd^ chromosome between alb267 and nas314 (Table 3). All the introgressed alleles are either hemizygous (*D1* and *D2*) or heterozygous (all the other loci). To avoid unnecessary complexities, we ignore the background nas314 allele in genotype nomenclatures throughout. When the introgressed alleles are made homozygous in some genotypes, both copies are included in the genotype nomenclature (*e*.*g*., S7 Fig).

**Table 3:** Summary of introgression studies: Phenotypes of HMS and SRD of various introgression lines.

# Genotype^1^	Sample size (n)	Offspring (f) (mean ± s.e.m.)	Sex ratio (k)^2^ (mean ± s.e.m.)
A. *D1D2D3D4*;*S3*	27	73.6 ± 11.5	0.823 ± 0.015
B. *D1D2D3D4*;*S4*	14	Total 1 male and 5 females	-
C. *D1D2D3D4*	67	Total 5 females only	-
D. *D1*;*S3S4*	18	101.1 ± 10.5	0.527 ± 0.014
E. *D1*;*S3*	45	110.0 ± 8.4	0.500 ± 0.007
F. *D1*;*S4*	43	20.4 ± 5.2	0.504 ± 0.018
G. *D1*	118	Total 3 females only	-
H. *D1D2D3*;*S3S4*	18	95.2 ± 10.9	0.821 ± 0.026
I. *D1D2D3*	29	No offspring	-
J. *D2D3D4*;*S3S4*	23	110.5 ± 11.8	0.692 ± 0.019
K. *D2D3D4*;*S3*	92	106.1 ± 3.6	0.707 ± 0.006
L. *D2D3D4*;*S4*	84	19.7 ± 2.4	0.799 ± 0.018
M. *D2D3D4*	85	2.8 ± 0.5	0.864 ± 0.029
N. *D2D3*;*S3S4*	24	147.3 ± 9.0	0.713 ± 0.023
O. *D2D3*	85	8.1 ± 1.4	0.766 ± 0.017
P. *D3D4*;*S3S4*	17	201.1 ± 12.2	0.679 ± 0.013
Q. *D3D4*;*S3*	18	134.0 ± 8.1	0.686 ± 0.024
R. *D3D4*	10	67.4 ± 20.6	0.703 ± 0.020
S. *D2*	28	68.5 ± 6.4	0.506 ± 0.013
T. *D4*	31	100.4 ± 8.2	0.500 ± 0.010
U. *S4*	33	99.8 ± 13.1	0.499 ± 0.009
V. *N*;*N* control^3^	108	114.9 ± 4.8	0.508 ± 0.004

^1^For all introgression genotypes, the distorters (*D1—D4*) and suppressors (*S3*, *S4*) are defined by two closely linked flanking markers of alb267. *D2* is hemizygous because it is on the X while *D3*, *D4*, *S3* and *S4* are all heterozygous; the other alleles of nas314 are not shown.

^2^Sex ratio summary statistics obtained by bootstrapping.

^3^All markers have the nas314 alleles.

We assayed the functions of *D1* by contrasting several genotype pairs (Table 4; S8 Fig). In some genetic backgrounds, *D1* had strong sterilizing effects while in others it had the dual functions of SRD and HMS. Similar contrasts were made for *D2*, *D3*, *D4*, *S3* and *S4*, as well as *S3* and *S4* together (*S3S4*). Each of these loci expressed both HMS and SRD from at least one contrast. The phenotypes of all these loci are obviously sensitive to genetic background. One illustrative example is the *R3* region. The shl2-hap1 allele at the *R3* region (*S1*) is a stronger SRD suppressor in Exp1 (Fig. 4), but its SRD suppressing effect was not detected in Exp3 (*HMS12*), apparently caused by the lack of strong distorter in the hybrids. Similarly, the HMS functions of *D2*, *D3* and *D4* were not detected in Exp1 but they were readily detected in some introgressions when alb267 alleles were put into a largely nas314 background. The varying penetrance might have reduced the power of QTL mapping as we noticed earlier. Colocalization of *D1* (*D5*) and *HMS1* (*HMS3*) was more evident by introgressions than QTL mappings (Table 4; *cf*. Fig. 4), so were the dual functions of the *S3/HMS6*, *S4/HMS7* and *HMS12* regions with additional introgressions. When we collected additional mapping data from introgression of shl2-hap1 into nas314 background, even the *HMS12* locus was readily detected to express SRD suppressing effect (S9 Fig)

**Table 4:** Summary of introgression studies: Evidence for the HMS and SRD functions of each locus.

Locus^1^	Phenotypes	Supportive Evidence	
		HMS (P value^2^)	SRD (P value^2^)
*D1*	- Strong HMS	B *vs* L, C *vs* M, I *vs* O U *vs* F (7.14 × 10^–9^)	-
	- Almost completely sterilizing by its own	G *vs* V	-
	- Dual HMS and SRD functions	A *vs* K (0.000625) H *vs* N (0.0246)	A *vs* K (2.0 × 10^–16^) H *vs* N (2 × 10^–16^)
*D2*	- HMS	S *vs* V (3.63 × 10^–6^) J *vs* P (1.03 × 10^–5^)	
	- Dual HMS and SRD functions	K *vs* Q (0.00923) M *vs* R (0.000199)	K *vs* Q (0.0476) M *vs* R (1.77 × 10^–6^)
*D3*	- SRD	-	R *vs* T (2 × 10^–16^)
	- Dual HMS and SRD functions	O *vs* S (5.8 × 10^–14^)	O *vs* S (2 × 10^–16^)
*D4*	- HMS	J *vs* N (0.0254)	-
	- Dual HMS and SRD functions	M *vs* O (0.0449)	M *vs* O (0.00177)
*S3*	- Strong rescuing effect on male sterility	A *vs* C, E *vs* G, Q *vs* R (0.0153), D *vs* F (1.75 × 10^–7^)	-
	- HMS rescuing and SRD suppressing	J *vs* L (1.16 × 10^–10^) K *vs* M (2 × 10^–16^)	J *vs* L (2.82 × 10^–14^) K *vs* M (5 × 10^–7^)
*S4*	- Rescuing effect on male sterility	F *vs* G, P *vs* Q (0.000621)	
	- HMS rescuing and SRD suppressing	L *vs* M (1.36 × 10^–7^)	L *vs* M (0.0189)
*S3S4*	- Rescuing effect on male sterility	D *vs* G, H *vs* I (0.000621), P *vs* R (0.000183)	-
	- HMS rescuing and SRD suppressing	J *vs* M (1.54 × 10^–13^) N *vs* O (4.8 × 10^–14^)	J *vs* M (1.04 × 10^–7^) N *vs* O (0.00471)

^1^The alb267 allele evaluated in nas314 background.

^2^Obtained by two-sample Wilcoxon test (HMS) or logistic regression (SRD).

The dual functions might be contributed by separate SRD and HMS genes, but the probability of the four HMS genes with negative effects are each colocalized with one distorter is only $4!\prod_{i=1}^{4}U_{i}$ = 0.0002, where $U_{i}$ is the 95% confidence interval for the *i*th QTL, as measured in a fraction of the X-3 chromosome (Materials and Methods). Similarly, the probability is 0.02 for the colocalizations of HMS and SRD genes in the *S3* and *S4* regions. The overall probability is only 4 × 10^–6^ if distinct genes control the dual functions in all six QTL intervals. This calculation, albeit rough, strongly argues that the dual functions are most likely to be pleiotropic effects of the same genes.

Notably, some escapers of sterile males sired nearly all female offspring (*e*.*g*., 13♀: 1♂ or k = 93%, Table 3), leading us to speculate that many sterile hybrid males may have been potentiated to express extreme SRD. We also assayed the dominance of *S3* by crossing *D2D3D4*;*S3* males and females to generate four types of offspring (S7 Fig). When *S3* had two copies in the background, the sex ratio was reduced to 0.601 of *D2D3D4;S3/S3* from 0.695 of *D2D3D4;S3*. When *S3* was absent, *D2D3D4* males can only sire an average of ∼3 males with sex ratio 0.864. Thus *S3* is a semidominant SRD suppressor (*cf*. ref. 27). This explains why this currently silenced SRD system can be reactivated if one complement of suppressors is absent, as clearly shown by SRD (k = 0.628) expressed in one BC1 genotype that differs from alb267 males by only one 2^nd^ chromosome (S10 Fig). It can also be inferred that the Y-3 chromosome of *D*. *albomicans* still hosts sensitive responder.

## Discussion

We have uncovered a cryptic SRD system within *D*. *albomicans* that appears to have a direct causal link to HMS and thus to speciation. This conclusion is based on the increasing genetic association of SRD and HMS with increasing mapping resolution. We reached the highest resolution in this study with a large collection of introgressions, yet we were unable to separate the SRD and HMS functions to distinct genes at all six major loci. It is much more likely that the dual functions of these loci are pleiotropic effects of the same genes.

Importantly, the phenotypes of distorters (*D1—D4*) and suppressors (*S3*, *S4*) follow the “conflict theory”: all distorters are X-linked and reduce male fertility while all suppressors are autosomal and increase male fertility. This is not the case between an “older” species pair *D*. *mauritiana* and *D*. *simulans* where the introgressed heterospecific alleles always augment male sterility regardless of their locations [7,19,32]. We interpret the male fertility functions of *D1—D4* vis-à-vis *S3* and *S4* as the former have primarily evolved as SRD distorters while the latter as suppressors. In chronological order, distorters might most likely evolve earlier than suppressors.

Under the above evolutionary scenario, the intraspecific variation of SRD and HMS genes between alb267 and shl2-hap1 can be interpreted in the following way: because Exp1 clearly shows that alb267 has stronger distorter (positive effects of the *D1-4* loci) but weaker suppressor (positive effect of the *S1-2* loci) (Exp1 of Fig. 4; S2 Table), the SRD system might have become cryptic in alb2 more recently than in shl2, while in the latter the distorter function has been degraded to become residual but the evolution of SRD left permanent footprint on spermatogenesis, so the HMS function such as that of the loci *HMS9* and *HMS12* stays. In light of this interpretation, the positive effect of *HMS1* in Exp1 no longer appear contradictory to the negative effects of *HMS3* in Exp2 and *HMS9* in Exp3, because the shl2-hap1 allele of *D1* expresses weaker SRD but stronger HMS than the alb267 allele. We must point out, however, comparing the magnitudes of HMS effects across HMS1, HMS3 and HMS9 might not be justified because they were measured in very different genetic backgrounds. Furthermore, the shl2-hap1 allele at the *R3* region has a stronger suppressing power on SRD than the alb267 allele (Exp1 in Fig. 4). These two different SRD suppressor alleles might also differ in their power to rescue male fertility. Taken together, the difference in HMS effects between alb267 and shl2 as observed earlier (Figs. 2 and 3) can be readily accounted by the SRD system divergence within *D*. *albomicans*.

Numerous so-called “speciation genes” have been identified by mapping and positional cloning in the last three decades but little evidence has been gathered for their roles in establishing the initial RI. Though studies suggest several DMI genes as relics of genomic conflicts [3], or indirectly implicate SRD as the primary evolutionary cause of DMI in fly [17,18] and mouse [33,34], this study is the first one to catch SRD in action and the first account that SRD is driving the evolution of most HMS-causing genetic divergence between two newly formed species. We have also shown the difficulty of simultaneously detecting both SRD and HMS phenotypes, because SRD expression is very sensitive to genetic background (Table 4). This difficulty might explain why so little empirical evidence has been accumulated so far for the “conflict theory” of speciation.

One QTL might contain multiple loci with less effect [32]; the dual functions might be caused by closely linked QTL each with one function only. Even if these possibilities turn out to be true under finer genetic analyses, a weaker version of the “conflict theory” could still be valid because HMS genes can hitchhike with the fixation of SRD genes. This possibility is best demonstrated in a classic case of RI between *Mimulus guttatus* ecotypes previously thought to be a pleiotropic by-product of adaptive evolution to copper contamination in soil. However, HI and copper tolerance are each controlled by tightly linked but distinct genes [35]. Unlike a scenario that RI is driven by ecological adaptation [36,37], the primary driving force emphasized by the “conflict theory” is intragenomic conflicts.

In a broader sense, our study sheds new light on the relationship between adaptive evolution—conventionally attributed to external biotic or abiotic factors—and speciation, which is generally regarded as a consequence of anagenesis under adaptive evolution [38]. Our study emphasizes that non-adaptive evolution out of intragenomic conflicts might be an important mechanism for evolution [39]. In addition to speciation, evolution of several biological traits might also be driven by intragenomic conflicts, such as mating behavior in some insects and epigenetic regulation of the sex chromosomes [13,40–42]. It would be extremely interesting to see how ubiquitous this mechanism would be in the evolution of many other biological traits.

The simplicity of the genetic architecture of SRD/HMS between *D*. *albomicans* and *D*. *nasuta* opens the door for future studies to fine map and positionally clone all key genes, and to study their population genetics and genomics as well as biogeography of the speciation process [43,44]. An elucidation of the mystery shrouding the speciation problem appears to be reachable at least for these two species.

Lastly, our study might help to address one long-standing controversy over the role of chromosomal rearrangement in speciation. Chromosomal rearrangements like Robertsonian fusions are often found among closely related species, thus are believed by some evolutionists to have played a major role in RI evolution because the F1 heterozygotes of two karyotypes are often less viable or fertile then the parents [45,46]. The difficulty of this theory is that the less fit heterozygotes would have prevented the new karyotype from spreading in a population, let alone founding new species [47]. *D*. *albomicans* with the fused X-3 chromosome has evolved from *D*. *nasuta*-like ancestor with separate X and 3^rd^ chromosome. Our study has shown that meiotic drive might indeed have helped the spread of the X-3 fusion and meiotic drive can play an important role in karyotype evolution. However, we have also shown that the current RI between *D*. *albomicans* and *D*. *nasuta* might actually be caused by genic factors, not necessarily by the chromosomal rearrangements *per se*. Therefore, we revise the original thesis on the role of chromosomal rearrangement in speciation to emphasize meiotic drive as the means to spread new karyotypes—as M. J. D. White speculated [46]—but karyotypic changes might not be directly causing RI.

## Materials and Methods

### *Drosophila* stocks and husbandry

Three inbred lines were constructed by sib pair matings for 15 generations from outbred stocks: *D*. *albomicans* alb2—from the strain E-10802/MYH01-05, Miyakojima, Okinawa, Japan, 2001; *D*. *albomicans* shl2—from the strain E-10815/SHL48, Shillong, India, 1981; and *D*. *nasuta* nas3—from the strain G86, Mauritius, 1979. Dr. M. Watada, Ehime University, Japan, kindly provided us these three stocks. For brevity, these three inbred stocks are named alb2, shl2 and nas3. These stocks were crossed to generate the F1, F2 and BC1 hybrids, which were tested for fertility and sex ratio and the result is consistent with previous work [24–27] (S1 Fig).

Based on polytene chromosomes and molecular markers (frequent double peaks in the Sanger sequencing chromatograms), we found all three stocks were still polymorphic for inversions. Multiple single pair matings were set up from alb2, nas3 and shl2. Inversion-free parents were identified based on sequencing select markers. We constructed the stocks alb267 and alb215, free of inversions, from alb2. We produced a standard, more accessible and better quality photograph polytene map from alb267, as compared to the same map published before [48] (S2 Dataset). Similarly, we constructed true-bred stocks nas314 and nas384 from nas3, with different polytene sequences on the third chromosome. We failed to construct true-bred stocks from shl2, presumably due to the recessive sterile mutations located in the inversions. All inversions in the three inbred stocks were identified based on polytenes prepared from various stocks and their hybrids, as summarized in Fig. 1.

Flies were reared on standard Cornmeal-Molasses-Agar food in plastic vials (ϕ2.6 × h9.4 cm). For all crosses, virgin tester females were aged to 5 days before setting up crosses at room temperature (22 ± 1°C).

### Polytene chromosome preparation

A pair of salivary glands were dissected out from a wandering third-instar larva—sex determined if necessary by the translucent gonads—in a drop of 45% acetic acid and quickly transferred to a second drop of 45% acetic acid for approximately 3 minutes. Individual glands were transferred to a drop of 2% lactic-acetic-orcein solution and stained for ∼5 minutes, then transferred again to a fresh drop of 2% lactic-acetic-orcein solution on a clean slide. The preparation was covered with a siliconized cover slip. The chromosomes were spread by gentle but firm tapping or pressing. The cover slips were sealed with nail polish. The preparations were stored up to 10 days at room temperature prior to examination under a 100× objective of an Olympus BX51 microscope. All cytological images were documented with an Olympus DP30BW digital camera. Further processing was done with Photoshop CS4 ver11.0.2.

### Molecular markers and genotyping

PCR primers of molecular markers were designed based on (1) cDNA sequences prepared from *D*. *albomicans* male [21] and (2) their alignments with the annotated homologs from *D*. *pseudoobscura*, *D*. *virilis* and *D*. *mojavensis* (http://flybase.org/). The predicted PCR products fall in the size range of 500–1000 bp and span intron(s) if possible. PCR products amplified from alb2, nas3 and shl2 were Sanger sequenced by Beckman Coulter Genomics (Danvers, MA).

Fixed nucleotide differences among stocks were used to develop allele-specific oligonucleotide (ASO) probes [6]. We have developed a total of 62 ASO markers between alb2 and shl2-hap1, 67 markers between alb267 and nas314, and 54 markers between shl2-hap1 and nas314. Many of these markers were also typed by restriction fragment length polymorphism (RFLP). Technical details of the probes, including PCR primers, ASO probes and wash temperatures, can be found in S1 Dataset.

To prepare DNA from single flies, an individual was quickly ground in a 1.5-ml Eppendorf tube with 200 μl extraction buffer (10 mM Tris pH 8.2, 1 mM EDTA, 25 mM NaCl, 0.4 mg/ml Proteinase K). After a 20-min digestion at 65°C, the tube was incubated at 95°C for 5 min and then chilled on ice. The extracted DNA was spun down briefly before being stored at -20°C.

PCR amplification was performed in a total volume of 10 μl reaction mixture (1× buffer, 0.2 μM forward and reverse primer mix, 0.25 units of *Taq* polymerase, 150 μM dNTP, and 1 μl DNA template). The amplified PCR products were genotyped by RFLP or ASO probes as previously described [6].

### Ultrastructural study of spermatogenesis by Transmission Electron Microscopy (TEM)

Testes and accessory glands were dissected out from young males (2–3 day old) with a fine tungsten needle and were transferred immediately to 2% glutaraldehyde in 0.067 M phosphate buffer on ice. The specimens were fixed for 2 hrs at 4°C in 1% paraformaldehyde and 2% glutaraldehyde in 0.067 M phosphate buffer, followed by a post fixation of 1 hr in 2% OsO4 at 4°C. The specimens were treated with 1% uranyl acetate at room temperature and were then dehydrated through ethanol grades (30% to 100%). Only one of each pair of testes was embedded.

Each testis was cut into 4–5 segments with a fine tungsten needle and these segments were then aligned on the bottom of a mold with the apical tip facing out to one end. Sections were cut on a Reichert ultracut-S microtome, followed by staining with uranyl acetate and lead citrate. The grids were observed with HITACHI H-7500 electron microscope at Emory University Apkarian IE Microscopy Core.

### Fertility and sex ratio assay

Two methods were used to measure fertility and sex ratio:

1. Standard method. Individual males (females) were crossed to 3 virgin females (males) in a vial for 7 days before the mating parents were discarded or kept for genotyping if necessary. The offspring were sexed and counted 4–5 times until the 19^th^ day after setup. Preliminary tests have shown that F1 hybrids between *D*. *albomicans* and *D*. *nasuta* produced normal or nearly normal numbers of progeny by this method (S1 Fig), as also suggested by previous work [24,25]. The carrying capacity of the food vial is ∼200 flies so the standard method might not be sensitive enough to measure slightly or even moderately reduced fertility.
2. Exhaustive mating protocol. We designated a method more sensitive than the standard one to quantify fertility. Throughout the experiments we used 5-day old virgin males or females of the same genotype (alb2) as the tester and controlled the temperature at 22 ± 1°C, because preliminary tests had shown that temperature and tester females had small but significant effects on male fertility of some genotypes (S1 Fig).

For male fertility assay, individual 1-day old males were mated to three tester females for 24 hrs (day 1). The males were subsequently transferred to fresh vials supplied with three virgin females on day 2 and day 3, after which the males were transferred to vials with 12 virgin females to stay in days 4–7 and then to vials with three virgin females for day 8. The 4 + 1 days transfer regime was repeated until the individual males were dead or sterile. To prevent crowding in vials the mated tester females in 1-day vials (days 1, 2, 3, 8, 13, *etc*.) were transferred to fresh vials every 7 days until they no longer laid fertilized eggs. To reduce the labor cost (by ∼80%) we only sexed and counted offspring from 1-day vials (days 1, 2, 3, 8, 13, *etc*.) to the 19^th^ day after vial setup, while the offspring from the 4-day vials (days 4–7, 9–12, *etc*.) were not counted and their numbers were interpolated from the flanking two 1-day vials, assuming the 4-day vials produced twice as many offspring from these two 1-day vials. Towards the end of the protocol, male fertility dropped to only a few offspring per day so all offspring were usually counted from both types of vials.

The above protocol for quantifying male fertility was designed under the assumptions that (1) all functional sperm fertilize eggs, and (2) the interpolation was accurate. In a pilot study we found male mating latency was more than 12 hrs, and the progeny size from the second mating within the same day was much smaller than the first mating. Therefore the first assumption is likely to be valid. The second assumption was shown to be valid also by two pilot experiments in which the alb2 and nas3 males were tested by the above protocol, with additional transfers of 4-day vial females to fresh vials and counting of their offspring. The actual counting and interpolation converges remarkably well (S3 Fig). Therefore the exhaustive mating protocol can be used to “count” the functional sperm produced by a male.

For female fecundity assay, single females were mated to three tester males in a vial and the flies were transferred to a fresh vial every 4 days until the female became sterile or died. Any dead male was replaced with fresh one during the experiment. All offspring were sexed and counted.

### QTL mapping

Because of the fixed inversions between alb267 (shl2-hap1) and nas314 on the 3^rd^ chromosome, ∼40% of the genome is refractory from meiotic mapping (Fig. 1). On the other hand, the two *D*. *albomicans* complements, alb267 and shl2-hap1, are homosequential so that meiotic mapping can cover the whole genome. With these considerations and to maximize the power of QTL mapping from the available lines, three QTL mappings were executed.

In the first QTL experiment (Exp1), we generated a population of 459 males by crossing individual F1 females (alb267/shl2-hap1 from alb267♀ × shl2♂) to nas314 males. After the vials were established, the mated F1 females of the genotype alb267/shl2-hap1 were distinguished from that of the genotype alb267/shl2-hap2 by molecular markers. Each male of the mapping population was phenotyped by crossing to alb2 females per standard method. These 459 males were genotyped for 62 ASO markers that can distinguish the alb267 and the shl2-hap1 alleles (S1 Dataset).

The other two QTL mappings were similarly executed. In Exp2, a population of 442 males was generated by backcrossing the F1 females from alb267♀ × nas314♂ to nas314 males, and was genotyped for 67 ASO markers. In Exp3, a population of 470 males was generated by crossing the F1 females (shl2-hap1/nas314 from shl2♀ × nas314♂) to nas314 males, and was genotyped for 39 ASO markers out of the 54 markers available because only three out of the 18 markers (M8, M24 and M30) on the non-recombining 3^rd^ chromosome were genotyped (S1 Table).

The phenotypes (male fertility and sex ratio) of all three QTL mapping populations are summarized in S4 Fig. Because it is not reliable to calculate sex ratio from small progeny size, we only use the males that sired at least 30 offspring. Thus the sample sizes of sex ratio for these three QTL mappings are reduced to 440, 227 and 340, respectively. Overall, males from Exp2 and Exp3 suffered much greater sterility than from Exp1, while SRD is almost absent from Exp3. This pattern is consistent with earlier observations that the F1 males from alb2♀ × nas3♂ and shl2♀ × nas3♂ were very infertile, while shl2 had only weak SRD alleles genes (Fig. 3).

We first applied the R/qtl package (v1.26) to construct three genetic maps separately from the three QTL mappings [49]. As expected, the 3^rd^ chromosome had normal recombination only in Exp1 but hardly any in the other two mappings (S5 Fig, S1 Table). M68 is not linked to the 2^nd^ linkage group in Exp2, while M41 and M45 are not informative in Exp3. In the end, the genetic maps of Exp2 and Exp3 are far less complete as compared to that of Exp1. Interestingly, there seems to be a cluster of markers around the centromere region on the 2^nd^ chromosome, suggesting the existence of chromosomal rearrangements in that area but we did not detect any polytene evidence for that suggestion.

To map QTL for HMS and SRD, we applied the composite interval mapping (CIM) method implemented in Windows QTL Cartographer (v2.5_008) [50,51]. Male fertility was treated as a continuous variable as either the raw counts (T) or transformed by log_10_^(T+1)^, or as a binary variable of 1 (fertile) and 0 (sterile), with different biological implications. For example, the difference between sterile (T = 0) and subfertile (say T = 10) is definitely more profound in terms of spermatogenetic defects than difference in fertility, say, of T = 100 and 110; thus the log_10_ or binary transformation might be closer to biological reality than the raw count T. Sex ratio (k) was also treated as continuous variable. The threshold for significant QTL was determined by 500 times of permutations of the datasets at the level α = 0.05.

The QTL mapping results are plotted in S5 Fig. The presence, location and magnitude of the HMS QTL are often sensitive to data transformation methods. We also applied the multiple interval mapping (MIM) method to evaluate the QTL flagged by CIM and the epistasis, if any, among them [52]. The total genetic components (*H* ^2^) and their additive parts (*h* ^2^) of all QTL were also obtained from MIM, as summarized in S2–S4 Tables. A synopsis of QTL mappings by different methods is presented in Fig. 4 and Tables 1 and 2.

### Introgression

For a much improved signal/noise ratio than that from QTL mapping, we thus wished to test the effects of the flagged individual QTL in a uniform and clean background. We used an introgression method to isolate a few chromosomal segments, each containing individual QTL, in a largely nas314 background including the Y chromosome. A typical scheme was shown in S6 Fig. The six QTL, the markers used to monitor their transmission and the approximate sizes of each interval (proportion of the X-3 or 2^nd^ chromosomes based on genetic distance) are: *D1* (M105 – M57, ∼3.7%), *D2* (M107 – M20, ∼3.8%), *D3* (M98 – M157, ∼11.1%), and *D4* (M30, ∼5.4%) on the X-3, *S3* (M72 – M136, ∼4.0%) and *S4* (M66 – M63, ∼25.5%) on the 2^nd^ chromosome. The estimated interval sizes might have large errors because of unequal cross-over frequencies along the chromosomes.

### Statistics

Sex ratio was treated as continuous variable with Gaussian distribution if all the progeny sizes were at least 30; otherwise logistic regression was applied on male and female counts. For summary statistics (mean and s.e.m.) of sex ratio obtained from sub-fertile males that often had progeny < 30, a bootstrapping method was used to avoid spurious results. Other methods were standard as indicated in the text.

## Supporting Information

S1 Fig Preliminary crosses among *D*. *albomicans* and *D*. *nasuta* stocks. Box plots of fertility and sex ratio measured by standard method from various crosses (♀ × ♂) using the three inbred lines of *D*. *albomicans* (alb2 and shl2) and *D*. *nasuta* (nas3). Crosses are shown below the abscissa while the sample sizes are shown above. Sex ratios were calculated from progeny ≥ 30 so their sample sizes are often smaller than that for fertility. (A, C) All crosses were set up at 25°C. The cause is unclear for the biased sex ratio (k = 0.69) found in the cross shl2 × (nas3 × shl2) but certainly not SRD expressed in the father (the F1 male from nas3 × shl2) because two other crosses (F1 × F1, nas3 × F1) with fathers of the same genotype did not produce biased sex ratio (C). On the other hand, sex ratio appeared to be normal in the progeny of the F1 males from shl2 × nas3 but weak SRD (k = ∼0.63) was detected in the same genotype by the exhaustive mating method (Fig. 3B). (B, D) Strong SRD expressed in the F1 males from alb2 × nas3 but not in the F1 males from the reciprocal cross. The fertility (T) of the F1 males from nas3 × alb2 was higher than that from the reciprocal cross (ΔT = 49 offspring/male, P << 0.001, linear regression). T was also positively correlated with temperature (+9.1 offspring/°C in the 18–25°C range, P << 0.001) (B). Tester females appeared to have small effect on progeny sex ratio because alb2 mothers produced higher progeny sex ratio than the F1 mothers (alb2 × F1 *vs*. F1 × F1, Δk = 0.034, P < 0.001). SRD was correlated to temperature, too (+0.003/°C, P = 0.02), but its expression at 22°C was similar to that at 25°C (P = 0.35) or 20°C (P = 0.55). For each cross, sex ratio deviation from 0.5 is marked at significance level. Only the F1 males from alb2 × nas3 consistently expressed high SRD (P < 0.05*, < 0.01** and < 0.001*** with Bonferroni correction, n = 39 total tests) (D).
(EPS)

S2 Fig Abnormal spermatogenesis found in some interspecific F1 males. TEM examination of cross sections at various stages of spermatogenesis in the F1 males from three interspecific (first three columns) and one intraspecific control crosses (♀ × ♂). (A) At young stage before pre-condensation of head, spermatid tails were normal in all but the F1 males from shl2 × nas3, which had slightly more frequent abnormalities such as missing mitochondrial derivatives (arrow head) (Mean ± s.e.m. presented underneath with numbers of bundles examined in parentheses. *** P < 0.001, ANOVA followed by TukeyHSD). (B) Mature sperm in seminal vesicle often had frequent twin fusions (arrow heads) of tails from the F1 males of alb2 × nas3 and shl2 × nas3 but not from the other two F1 males. (C) In contrast, sperm heads at condensation stage were normal for all genotypes. All scale bars are 1 μm.
(TIF)

S3 Fig The exhaustive mating protocol for quantifying male fertility. In two pilot experiments with alb2 (A) and nas3 males (B), the tested males were offered virgin females on a 4+1 day transfer regime according to the exhaustive mating protocol (see Materials and Methods for details). In addition to counting progeny from the 1-day vials, the 4-day vials were also counted. Note that male fertility dropped to a few progeny per day towards the end of the test, so all the progeny from the last few transfers were also counted. The actual counts and the interpolations from the two flanking 1-day vials are remarkably consistent. Error bar represents 1× s.e.m. n: sample size. Numbers within the graph: time points (day) of transfer.
(EPS)

S4 Fig Distributions of male fertility and sex ratio in the three QTL mapping populations. (A) Male fertility (T) was standardized by T/maximum T × 100, making comparison across experiments easier. (B) Sex ratio (k) was calculated from progeny ≥ 30. n: sample size.
(EPS)

S5 Fig Three QTL mappings of HMS and SRD. In QTL analysis, male fertility was treated in 3 ways: as progeny size (T), as a transformed quantity log_10_^(T+1)^, or as a binary of 1 for fertile and 0 for sterile. Sex ratio (k) was calculated from males with T ≥ 30. The QTL were mapped by CIM and the 5% thresholds were computed by 500 permutations. All thresholds are similar so only one is shown. (A) Exp1, mapping between alb267 and shl2-hap1. (B) Exp2, mapping between alb267 and nas314. M68 is not linked to Chr. 2. The location and contribution of either *HMS7-1* or *HMS7-2* are uncertain. (C) Exp3, mapping between shl2-hap1 and nas314. *HMS9-2* is probably not a real QTL (S11 Fig). Note the different scale of ordinates (LOD) for sex ratio (orange) and fertility. More details of the linkage groups and the relevant statistics of QTL mapping are summarized in S1–S4 Tables. In this figure, the length for each major linkage group is given at the end.
(EPS)

S6 Fig The introgression scheme. Chromosomal segments of alb267 were introgressed into the nas314 background with the assistance of markers (m1-m6). Any introgression males can be used to start the scheme to generate new recombinants through females (G1 and G2), so shorter introgressions (*e*.*g*., T2, compared to T1) can be screened in G3 (*e*.*g*., *s2* derived from *s1*), and new genotype (*e*.*g*., T3: *d*;*s2*) can be further generated from G3 to G5. The phenotypic differences between different genotypes can be used to map genes. For example, alb2 allele in the m5-centromere-m6 region can be inferred to underlie the male fertility difference between T1 and T3. The X, Y, 2nd and 3rd chromosomes are labeled as X, Y, 2 and 3.
(EPS)

S7 Fig Suppressor *S3* is semidominant as tested in introgressions. To test the dominance of *S3*, the *D2D3D4;S3* males and females were crossed to generate four genotypes of offspring: *N;S3*, *N;S3/S3*, *D;S3* and *D;S3/S3*, where *N* = nas3 allele at all of the *D2–D4* loci and *D* = *D2D3D4* (A). The sex ratio of *D;S3/S3* is much lower than *D;S3* but higher than the controls *N;S3* and *N;S3/S3* (ANOVA followed by TukeyHSD) (B). For brevity, the genotype nomenclature denotes only the alb267 allele if one locus is heterozygous. For homozygous introgressions, both alleles are included.
(EPS)

S8 Fig Examples of individual QTL with dual functions of HMS and SRD. All tested genotypes were introgressions of the alb267 alleles into the nas314 background. (A) *D1*, shown here are male and female offspring sired by males of *D1D2D3D4;S3* contrasted to that of *D2D3D4;S3*. (B) *D2* (*D2D3D4* vs. *D3D4*). (C) *D3* (*D2D3* vs. *D2*). (D) *S3* (*D2D3D4;S4* vs. *D2D3D4;S3S4*). For each genotype, the sample size is shown in parenthesis, and the summary statistics (mean ± s.e.m.) for sex ratio is obtained by bootstrapping. The offspring counts are scaled by log_10_ but sterile (0) is arbitrarily positioned. The diagonal line represents equal sex ratio. If the same counts of males and females were observed for multiple times, larger markers are used with their areas scaled proportionally (D). *** P < 0.001, Wilcoxon test for fertility, and logistic regression for sex ratio.
(EPS)

S9 Fig Dual functions of SRD and HMS are shown by additional introgressions. Additional introgressions of the alb267 or shl2-hap1 alleles into the nas314 background were tested for male fertility and sex ratio. For comparison, QTL mapping results on the 2^nd^ chromosome from Exp2 and Exp3 are also shown (see Fig. 4), after the graphs being rescaled to roughly align the same markers between the two experiments. (A) Introgressions of the alb267 alleles into the nas314 background. These lines are grouped into three genotype classes: (I) *D2D3D4;S3*(*HMS6*), (II) *D2D3D4;S4*(HMS7) and (III) *D2D3D4;HMS8*. The tentative QTL *S3* and *HMS7* are evidently confirmed by the introgression genotypes I and II, respectively. The effect of *HMS8* is also somewhat confirmed by comparing genotype III to *D2D3D4* (Table 3) (male fertility: f = 8.4 ± 1.7 *vs*. 2.8 ± 0.5, P = 0.0009), with the caveat that the experiments were executed in different times so the results might be compounded with genetic and environmental background. QTL mapping positioned *HMS6* to the interval of 24.8–28.6 with 95% confidence, at the right side of M81 (24.59, S1 Table), but its position is clearly at the left of M81 by comparing I.1, I.2 and II.1. Within genotype class II, fertility seems to increase with longer introgressions, suggesting an HMS factor (*) between M136 and M127 but its effect is too weak to detect confidently. Both *HMS7* and *S4* are narrowed down to between M127 and M142. *HMS6* and *S3* are likely the same locus, so are *HMS7* and *S4*. (B) Introgressions of the shl2-hap1 alleles into the nas314 background on the 2^nd^ chromosome, with the distorters *D2D3D4* from alb267 and shl2-hap1, respectively. The genotype X is *D2D3D4* with no shl2-hap1 allele present on the 2^nd^ chromosome. *HMS12* was positioned in the region of 104.8–107.7 in QTL mapping Exp3, but it must be at the right of M77 (106.7) and has sex ratio suppressing effect as clearly demonstrated by comparing the genotypes VIII and IX to X. However, *S6* might be too weak to declare because the genotype IV has similar phenotypes as *D2D3D4* (P = 0.219 for f, and P = 0.998 for k). The weak locus (*) located to the M136—M127 interval might be real because the genotype V did have better fertility than *D2D3D4* (f = 6.8 ± 1.2 *vs*. 2.8 ± 0.5, P = 0.00178). Some SRD or HMS genes from alb267 and shl2-hap1 might be allelic (double headed arrow). Overall, the introgressions provide additional evidence with better power and resolution to support the hypothesis that major SRD and HMS genes are often colocalized, possibly to the same loci. Statistics—All summary statistics were obtained by bootstrapping. For fertility, offspring rankings were analyzed with ANOVA and TukeyHSD. For sex ratio, counts of male and female offspring were analyzed with logistic regression, and then processed with the glht function from the R/multcomp package in lieu of TukeyHSD. Male fertility (f) and sex ratios (k) not sharing the same superscripts are significantly different (P < 0.05).
(EPS)

S10 Fig The cryptic SRD system in *D*. *albomicans* is reactivated when one copy of suppressor is removed. Individual BC1 males from the cross alb2♀ × (nas3♀ × alb2♂)♂ had been mating-tested by the standard method before they were collected and genotyped for the 2nd and 4^th^ chromosomes, which were either alb2/nas3 or alb2/alb2. Sex ratio expressed in the former genotype was significantly higher than that in the latter one (k = 0.628 *vs*. 0.520, P < 2.2 × 10^–16^, logistic regression). The 4^th^ chromosome had no effect on sex ratio. SRD is uncovered in alb2-like males with only one copy of the 2^nd^ chromosome replaced by one of nas3, suggesting that the cryptic SRD system in *D*. *albomicans* still have active distorter, sensitive responder and effective suppressor.
(EPS)

S11 Fig *HMS9-2* is not a real QTL. (A) Schematic of the introgression line S1.1. The break points distal to M105 and in the M56 – M107 region are not determined. *HMS9-1* is located between M105 and M57, while *HMS9-2* is located between M57 and M50 (S6C Fig). (B) Boxplots of male fertility for the two types of recombinants (*NNS* and *SSN*) and the parental types (*NNN* and *SSS*). Sample sizes are shown in boxes. The genotypes stand for alleles at three markers (M105-M57-M56) (*S*—shl2-hap1; *N*—nas314). All males were tested as *X* ^introgression^/*Y* ^nas314^; *3* ^nas314^/*3* ^nas314^; *2* ^introgression^/*2* ^nas314^ where *2* ^introgression^ is an introgression of the shl2-hap1 alleles into the nas314 background spanning at least from M83 to M127 (S1 Table and Fig. 4). Because there is no fertility difference between *NNN* and *NNS*, as well as between *SSN* and *SSS* (ANOVA using fertility ranking, both P > 0.05), the effect of *HMS9-2* is not significant and is thus lumped with *HMS9-1* together as a single locus *HMS9*.
(EPS)

S1 Dataset Molecular markers used in this study. (XLSX)

S2 Dataset Standard polytene map of *D*. *albomicans*. This map was made from alb267, which has the same band sequence and nomenclature as one of the published maps [48].
(EPS)

S1 Table Linkage groups (LGs) obtained from the three QTL mappings. (PDF)

S2 Table Summary of QTL mappings: Exp1. (PDF)

S3 Table Summary of QTL mappings: Exp2. (PDF)

S4 Table Summary of QTL mappings: Exp3. (PDF)
