# Supplementary figures and images for: Sex Ratio Meiotic Drive as a Plausible Evolutionary Mechanism for Hybrid Male Sterility
Source: PLoS Genet. 2015 Mar 30;11(3):e1005073. doi: 10.1371/journal.pgen.1005073 (PMC4379000; doi:10.1371/journal.pgen.1005073)

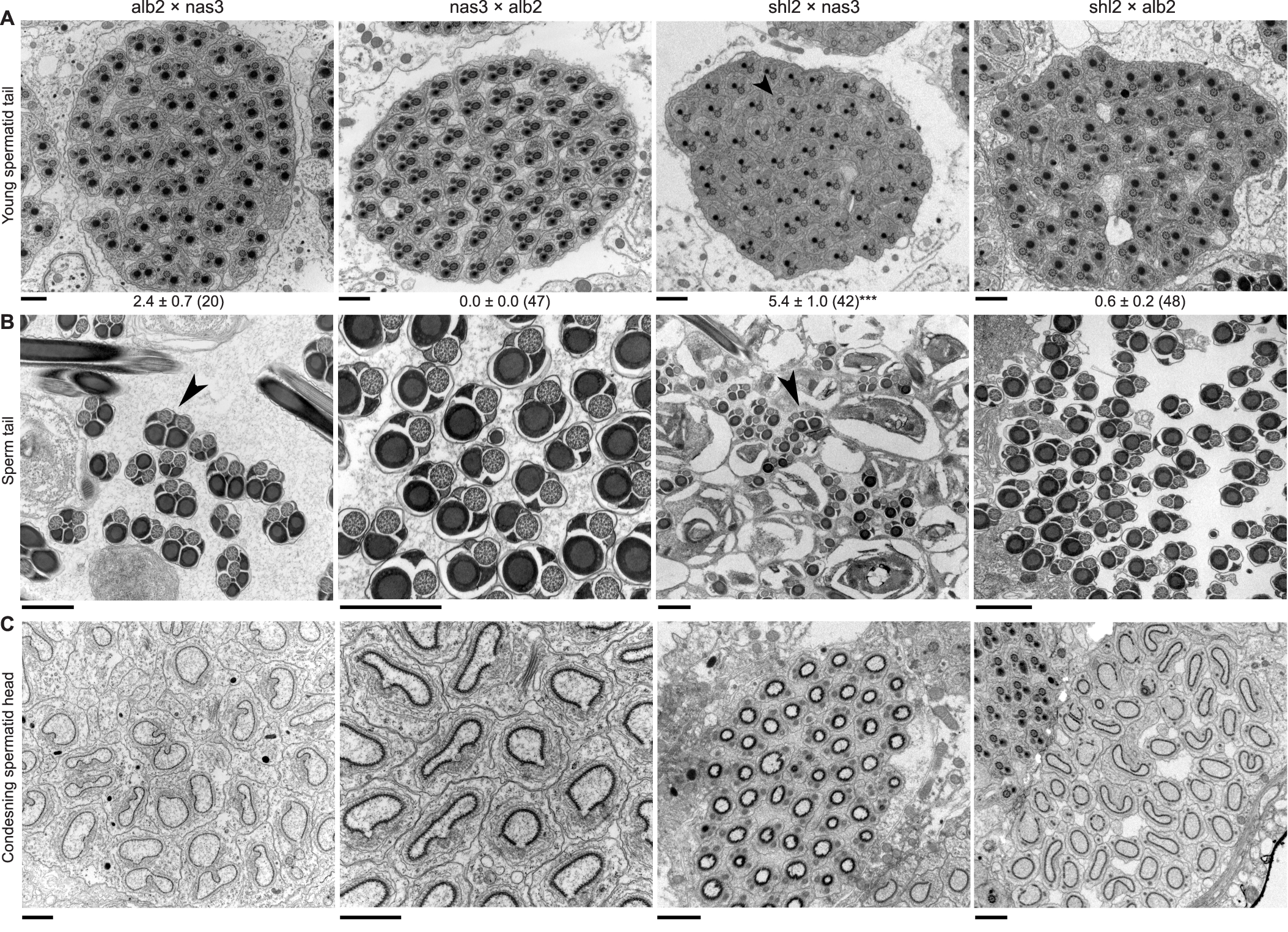

Supplement: S2 Fig — TEM examination of cross sections at various stages of spermatogenesis in the F1 males from three interspecific (first three columns) and one intraspecific control crosses (♀ × ♂). (A) At young stage before pre-condensation of head, spermatid tails were normal in all but the F1 males from shl2 × nas3, which had slightly more frequent abnormalities such as missing mitochondrial derivatives (arrow head) (Mean ± s.e.m. presented underneath with numbers of bundles examined in parentheses. *** P < 0.001, ANOVA followed by TukeyHSD). (B) Mature sperm in seminal vesicle often had frequent twin fusions (arrow heads) of tails from the F1 males of alb2 × nas3 and shl2 × nas3 but not from the other two F1 males. (C) In contrast, sperm heads at condensation stage were normal for all genotypes. All scale bars are 1 μm. (TIF) [file pgen.1005073.s002.tif]
