# Supplementary material for: Sex Ratio Meiotic Drive as a Plausible Evolutionary Mechanism for Hybrid Male Sterility
Source: PLoS Genet. 2015 Mar 30;11(3):e1005073. doi: 10.1371/journal.pgen.1005073 (PMC4379000; doi:10.1371/journal.pgen.1005073)
Supplement: S1 Table — (PDF) [file pgen.1005073.s014.pdf]

S1 Table. Linkage Groups (LG) obtained from the three QTL mappings

LG1	Muller's Elements	Exp1	Exp2	Exp3
M41	A	0.00	0.00	x
M45	A	0.00	0.00	x
M105	A	23.75	38.25	0.00
M57	A	38.62	52.70	22.60
M110	A	43.33	58.77	x
M50	A	64.97	74.12	59.35
M56	A	70.68	78.65	65.83
M44	A	72.82	81.93	69.32
M14	A	72.82	81.93	69.32
M108	A	90.91	98.67	88.30
M107	A	112.47	115.40	103.82
M20	A	133.97	130.24	123.26
M8	C	133.97	130.24	123.26
M24	C	135.80	130.24	123.26
M151	C	138.02	130.24	NT
M153	C	141.00	130.24	NT
M69	C	149.00	130.24	NT
M98	C	167.13	130.24	NT
M157	C	180.18	130.24	NT
M99	D	181.79	130.24	NT
M26	C	184.95	130.24	NT
M160	D	192.37	130.24	NT
M51	A	193.79	130.24	NT
M29	D	205.20	130.24	NT
M113	D	207.26	130.24	NT
M30	D	212.08	130.01	123.26
M119	D	212.08	130.01	NT
M102[1]	C	x	130.24	NT
M96[1]	D	x	130.24	NT
M158[1]	D	x	130.24	NT

LG3		Exp1	Exp2	Exp3
M36	F	0	0	0

LG4		Exp1	Exp2	Exp3
M68	B		0	

LG2	Muller's Elements	Exp1	Exp2	Exp3
M68	B	0.00		0.00
M88	B	46.02	0.00	69.20
M87	E	46.02	0.00	69.20
M83	B	x	7.08	79.46
M76	B	53.66	7.08	79.46
M89	B	55.33	12.52	84.44
M71	B	60.45	17.04	91.83
M73	B	63.11	18.63	96.25
M3	B	63.11	19.04	x
M82	B	64.21	21.63	100.21
M2	B	64.84	22.33	x
M77	B	67.11	24.36	106.65
M72	B	68.20	24.36	108.56
M43	B	68.20	24.36	x
M21	B	68.20	24.36	108.56
M146	E	68.20	24.36	x
M132	E	68.20	24.36	x
M133	E	68.20	24.36	x
M81	B	68.52	24.59	x
M148	E	68.73	24.81	x
M125	E	69.33	25.50	x
M136	E	x	26.51	113.46
M66	E	73.05	33.49	123.13
M127	E	75.20	36.33	128.25
M138	E	78.62	39.80	132.94
M134	E	83.23	47.26	140.19
M129	E	83.23	47.26	140.19
M63	E	83.44	47.26	140.19
M142	E	83.44	47.26	140.19
M150	E	102.55	67.64	170.17
M65	E	108.47	72.75	178.10
M141	E	109.94	73.54	x
M149	E	111.43	76.08	183.98
M122	E	117.16	81.87	194.88
M62	E	117.16	81.87	194.88
M33	E	117.16	89.04	194.88

		Exp1	Exp2	Exp3
#total		62	67	39

Positions are in cM.
x - no marker available.
NT- marker available but not typed.
[1]The positions of these three markers on the 3rd chromosome are unknown.
