# Supplementary material for: Sex Ratio Meiotic Drive as a Plausible Evolutionary Mechanism for Hybrid Male Sterility
Source: PLoS Genet. 2015 Mar 30;11(3):e1005073. doi: 10.1371/journal.pgen.1005073 (PMC4379000; doi:10.1371/journal.pgen.1005073)
Supplement: S2 Table — (PDF) [file pgen.1005073.s015.pdf]

S2 Table. Summary of QTL mappings: Exp1

Analysis and method			Chr.	QTL	CIM					MIM				
Phenotype	LOD threshold	Map. pop.			Position	CI-L[1]	CI-R[1]	Effect	H^2(%)	Position	LOD	Effect	H^2 (%)	h^2 (%)
Offspring (T)	2.4	499	X-3	*HMS1*	36.8	32.5	40.6	22.5	17.6	36.8	19.1	22.9	18.2	90.1
			2	*HMS2*	47.1	22.4	54.9	-8.0	9.3	48.0	2.4	-7.8	2.0	9.9
			**Sum**						**26.9**				**20.2**	**100**
Log_{10} (T+1)	2.2	459	X-3	*HMS1*	38.6	34.0	40.6	0.089	12.6	36.8	15.8	0.097	15.5	88.6
			2	*HMS2*	49.0	47.3	54.4	-0.059	3.4	47.0	2.4	-0.036	2.0	11.4
			**Sum**						**16.0**				**17.5**	**100**
Sex ratio (k)	2.1	440	X-3	*D1*	40.6	31.7	43.3	0.028	4.96	40.6	8.01	0.028	4.5	9.9
				D2	129.5	121.2	135.5	0.038	8.92	129.5	12.34	0.038	12.4	27.4
				D3	179.1	173.4	180.9	0.040	8.04	179.1	12.29	0.040	15.8	34.9
				D4	211.3	205.7	R-tip	0.022	2.53	211.3	4.54	0.028	6.5	14.3
			2	*S1*	63.1	60.3	64.8	0.025	4.20	63.1	5.49	0.023	4.7	10.4
				S2	-	-	-	-	-	114.4	1.43	0.012	1.4	3.1
				D1 × *D2*							3.2	0.018	2.4	
			**Sum**						**28.6**				**47.7**	**100**

[1] CI-L and CI-R: the left and right positions of the 95% confidence intervals
