# Supplementary material for: Sex Ratio Meiotic Drive as a Plausible Evolutionary Mechanism for Hybrid Male Sterility
Source: PLoS Genet. 2015 Mar 30;11(3):e1005073. doi: 10.1371/journal.pgen.1005073 (PMC4379000; doi:10.1371/journal.pgen.1005073)
Supplement: S3 Table — (PDF) [file pgen.1005073.s016.pdf]

S3 Table. Summary of QTL mappings: Exp2

Analysis and method			Chr.	QTL	CIM					MIM				
Phenotype	LOD threshold	Map. pop.			Position	CI-L[1]	CI-R[1]	Effect	H^2 (%)	Position	LOD	Effect	H^2 (%)	h^2 (%)
Offspring (T)	1.9	442	X-3	*HMS3*	51.3	44.1	56.1	-11.5	6.0	51.3	10.1	-12.5	8.1	20.2
				HMS4	-	-	-	-	-	98.7	1.3	-4.7	2.6	6.5
				HMS5	129.4	120.6	130.0	-6.9	1.9	129.4	3.3	-7.4	3.5	8.7
			2	*HMS6*	25.5	24.8	28.6	19.8	17.9	25.5	19.3	18.2	19.9	49.6
				HMS8	59.3	47.2	70.7	8.1	2.8	59.3	3.3	8.1	6.0	15.0
				HMS3 × *HMS4*							1.9	5.3	1.0	
				HMS3 × *HMS6*							2.1	5.2	1.2	
			**Sum**						**26.9**				**42.3**	**100**
Log$_{10}$ (T+1)	2.2	459	X-3	*HMS3*	49.3	43	54.8	-0.2	6.3	46.3	27.7	-0.28	10.6	16.8
				HMS4	95.9	83.2	109.8	-0.1	1.2	90.9	13.5	-0.21	7.4	11.7
				HMS5	129.4	120.7	130	-0.1	1.2	129.4	4.5	-0.09	2.1	3.3
			2	*HMS6*	25.5	24.6	29.1	0.4	15.9	28.5	44.0	0.37	28.8	45.6
				HMS7-1	37.3	37.3	38.4	0.5	37.2	-		-	-	
				HMS8	51.3	47.3	60.6	0.2	4.2	53.3	16.5	0.23	14.2	22.5
				HMS3 × *HMS4*							16.6	0.22	6.8	
				HMS4 × *HMS5*							2.5	- 0.069	1.0	
				HMS3 × *HMS6*							20.1	0.20	5.0	
				HMS5 × *HMS6*							1.4	0.050	1.3	
				HMS4 × *HMS8*							11.6	0.20	6.2	
				HMS6 × *HMS8*							6.7	0.14	2.0	
			**Sum**						**66.0**				**85.4**	**100**
Binary (T)	2.0	442	X-3	*HMS3*	47.3	41.5	52.6	-0.1	6.0	47.3	30.4	-0.13	8.8	14.5
				HMS4	105.7	96.7	112.7	-0.1	4.2	105.7	47.6	-0.12	5.7	9.4
			2	*HMS6*	24.8	24.3	26.4	0.2	13.4	23.3	17.6	0.098	12.8	21.1
				HMS7-2	42.8	41.2	45.8	0.2	10.4	43.8	21.8	0.25	33.5	55.1
				HMS3 × *HMS4*							48.1	0.13	7.4	
				HMS3 × *HMS6*							24.2	0.13	6.6	
				HMS4 × *HMS6*							16.3	-0.11	-4.8	
				HMS3 × *HMS6*							1.9	- 0.029	-1.0	
				HMS4 × *HMS7-2*							50.8	0.25	22.6	
			**Sum**						**34.0**				**91.6**	**100**
Sex ratio (k)	2.0	227	X-3	*D5*	54.7	44.2	72.9	2.4	1.4	52.7	0.3	0.015	1.8	2.8
				D6	130.0	129.9	Rt tip	14.2	54.2	130.0	29.6	0.13	49.1	76.7
			2	*S3*	-	-	-	-	-	18.6	2.4	0.037	5.2	8.1
				S4	44.8	38.8	52	5.1	4.7	43.8	6.2	0.052	7.9	12.3
				D5 × *S3*							1.3	0.032	1.6	
				D6 × *S4*							2.6	0.040	12.4	
			**Sum**						**60.3**				**78.0**	**100**

[1]CI-L and CI-R: the left and right positions of the 95% confidence intervals
