# Supplementary material for: Sex Ratio Meiotic Drive as a Plausible Evolutionary Mechanism for Hybrid Male Sterility
Source: PLoS Genet. 2015 Mar 30;11(3):e1005073. doi: 10.1371/journal.pgen.1005073 (PMC4379000; doi:10.1371/journal.pgen.1005073)
Supplement: S4 Table — (PDF) [file pgen.1005073.s017.pdf]

S4 Table. Summary of QTL mappings: Exp3

Analysis and method			Chr	QTL	CIM					MIM				
Phenotype	LOD threshold	Map. pop.			Position	CI-L[1]	CI-R[1]	Effect	H^2 (%)	Position	LOD	Effect	H^2 (%)	h^2 (%)
Offspring (T)	2.1	470	X-3	*HMS9-1*	17.0	14.5	19.1	-120.4	60.2	15.0	20.3	-84.4	48.0	55.9
				HMS9-2	28.6	25.9	31.3	-112.9	39.8	30.6	7.8	-53.1	28.1	32.7
				HMS10	80.3	72.1	87.3	-23.6	2.4	76.3	2.3	-11.2	2.4	2.8
			2	*HMS12*	106.7	104.8	107.7	45.3	9.3	106.7	29.9	35.4	6.6	7.7
				HMS14	192.0	183.8	193.0	14.2	0.9	192.0	3.2	11.7	0.8	0.9
			Sum						112.7				86.9	100
Log_{10} (T+1)	2.1	470	X-3	*HMS9-1*	18.0	14.8	19.9	-84.4	66.2	18.0	-2.2	-0.691	53.7	66.8
				HMS9-2	24.6	22.6	27.3	-68.3	28.9	24.6	3.4	-0.209	14.5	18.0
				HMS11	122.8	115.5	122.83	10.5	1.0	122.8	7.3	0.099	0.20	0.2
			2	*HMS12*	111.6	110	113.4	36.6	12.7	107.7	70.9	0.343	10.7	13.3
				HMS13	170.2	154.3	175.1	11.7	1.3	170.3	9.0	0.109	1.3	1.6
				HMS9-1 × *HMS9-2*							2.4	-0.132	0.6	
				HMS9-1 × *HMS11*							19.4	0.165	2.3	
				HMS9-1 × *HMS12*							48.6	0.258	4.9	
				HMS11 × *HMS12*							10.9	0.117	0.9	
				HMS9-1 × *HMS13*							9.5	0.110	1.6	
			**Sum**						**110.1**				**90.7**	**100**
Binary (T)	2.0	470	X-3	HMS9-1	19.0	14.8	21.4	-27.0	40.9	19.0	149.7	-0.307	47.6	70.4
				HMS11	122.8	114.83	122.83	6.6	2.5	122.8	12.2	0.056	1.1	1.6
			2	HMS12	112.6	110.5	116.6	16.3	15.1	112.6	106.2	0.191	17.3	25.6
				HMS13	170.2	155.4	176.7	5.5	1.7	170.3	10.1	0.046	1.6	2.4
				HMS9-1 × *HMS11*							10.8	0.054	1.5	
				HMS9-1 × *HMS12*							103.3	0.187	17.9	
				HMS11 × *HMS12*							10.4	0.048	0.9	
				HMS9-1 × *HMS13*							8.9	0.045	1.9	
				HMS11 × *HMS13*							5.5	-0.032	0.5	
			**Sum**						**60.2**				**90.3**	**100**
Sex ratio (k)	2.0	340	X-3	*D7*	-	-	-	-	-	19.0	0.45	-0.006	0.7	5.3
				D8	122.8	115.43	122.83	0.0183	9.9	123.0	1.6	0.010	5	38.2
			2											
				S5	20.0	0	51.9	-0.0126	5.2	6.0	2.0	-0.010	3.2	24.4
				S6						131.2	3.4	0.013	2.6	19.8
				S7						170.3	1.3	-0.008	1.6	12.2
			**Sum**						**15.1**				**13.1**	**100**

[1]CI-L and CI-R: the left and right positions of the 95% confidence intervals
